# Methylation of *miR-34a, miR-34b/c*, *miR-124-1 *and *miR-203 *in Ph-negative myeloproliferative neoplasms

**DOI:** 10.1186/1479-5876-9-197

**Published:** 2011-11-14

**Authors:** Chor Sang Chim, Thomas S Wan, Kwan Yeung Wong, Tsz Kin Fung, Hans G Drexler, Kit Fai Wong

**Affiliations:** 1 Department of Medicine, Queen Mary Hospital, The University of Hong Kong, Hong Kong; 2 Department of Pathology, Queen Mary Hospital, The University of Hong Kong, Hong Kong; 3 Department of Human and Animal Cell Cultures, DSMZ - German Collection of Microorganisms and Cell Cultures, Inhoffenstr. 7B 38126 Braunschweig, Germany; 4 Department of Pathology, Queen Elizabeth Hospital, Hong Kong

**Keywords:** microRNA, tumor suppressor, hypermethylation, Ph-negative myeloproliferative neoplasm

## Abstract

**Background:**

MicroRNA (miR) *miR*-*34*a, -*34b/c*, -*124-1 *and -*203 *are tumor suppressor miRs implicated in carcinogenesis.

**Methods:**

We studied DNA methylation of these miRs in Philadelphia-negative (Ph-ve) myeloproliferative neoplasms (MPNs). Methylation-specific PCR (MSP), verified by direct sequencing of the methylated MSP products, was performed in cell lines, normal controls and diagnostic marrow samples of patients with MPNs.

**Results:**

Methylation of these miRs was absent in the normal controls. *miR-34b/c *were homozygously methylated in HEL cells but heterozygously in MEG-01. In HEL cells, homozygous *miR-34b/c *methylation was associated with miR silencing, and 5-aza-2'-deoxycytidine treatment led to re-expression of both *miR-34b *and *miR-34c*, consistent with that both miRs are under the regulation of the same promoter CpG island. *miR-34a *was heterozygously methylated in MEG-01 and K-562. *miR-203 *was completely unmethylated in K-562 and SET-2 but no MSP amplification was found in both HEL and MEG-01, suggestive of miR deletion. In primary samples, four each had *miR-34b/c *and *-203 *methylation, in which two had concomitant methylation of *miR-34b/c *and *-203*. *miR-34a *was methylated in one patient and none had methylation of *miR-124-1*. Seven patients (15.6%) had methylation of at least one of the four miRs. miR methylation did not correlate with clinical parameters, disease complications or JAK2 V617F mutation.

**Conclusion:**

This is the first report of miR hypermethylation in MPNs. *miR-203 *hypermethylation is not specific to Ph+ve leukemias but also present in Ph-ve MPNs. *miR-34b/c *methylation was associated with reversible miR silencing. There was no correlation of miR methylation with clinical demographic data or outcome.

## Background

Philadelphia-negative (Ph-ve) myeloproliferative neoplasm (MPN) is a stem cell disease with proliferation of myeloid lineage, leading to the development of distinct clinical entities including polycythemia vera (PV), essential thrombocythemia (ET) and primary myelofibrosis (PMF) [1-3]. JAK2 V617F mutation, resulting in constitutive activation of JAK-STAT signaling, occurs in about half of the patients with ET and PMF but in more than 90% of patients with PV [1].

Gene methylation is an alternative mechanism of gene inactivation, and various tumor suppressor genes regulating the cell cycle, apoptosis and cell signaling have been shown to be hypermethylated in hematological malignancies [4].

MicroRNA (miR) is a single-stranded, non-coding RNA molecule of 22-25 nucleotides, which leads to downregulation of target protein expression [5]. miRs are involved in carcinogenesis [6]. miRs can be either oncogenic (oncomir) when tumor suppressor genes (TSG) are targeted, or tumor suppressive (tumor suppressor miRs) when oncogenes are targeted [7].

Recently, *miR-34a, miR-34b/c, miR-124-1 *and *miR-203 *hypermethylation have been implicated in carcinogenesis. Hypermethylation of *miR-34a*, a transcriptional target of p53, has been demonstrated in solid and hematopoietic cancers [8,9], whereas restoration of which will inhibit CDK6 translation by complementary binding to the 3' untranslated region (3' UTR) of the *CDK6 *mRNA and induce apoptosis, thereby showing the tumor suppressor role of *miR-34a *[8]. Epigenetic inactivation of *miR-34b*, another p53 downstream target of the *miR-34 *family, has also been implicated in acute myeloid leukemia (AML), and the re-expression of *miR-34b *led to suppression of CREB expression and inhibition of cell proliferation [10]. Promoter methylation of *miR-124-1*, the first tumor suppressor miR found to be regulated by DNA methylation, has been shown to confer poor prognosis of acute lymphoblastic leukemia (ALL) [11]. Moreover, hypermethylation of *miR-203 *has been reported in chronic myeloid leukemia (CML), conferring a proliferative advantage to the tumor cells by inhibiting the oncogenic BCR-ABL fusion protein [12]. In Ph-ve MPN, little is known about the epigenetic alteration of miR methylation. In this report, we studied the methylation status of *miR-34a, miR-34b/c, miR-124-1 *and *miR-203 *in PV, ET and PMF.

## Methods

### Patient samples

DNA was extracted from primary marrow samples at diagnosis of 45 patients with MPN [ET, N = 34 (75.5%); PV, N = 8 (17.8%) and PMF, N = 3 (6.7%)]. There were 24 (53.3%) male and 21 (46.7%) female patients with a median age of 67.5 years (range: 28 - 89 years), a median presenting platelet count of 848 × 10^9^/L (range: 196 - 2275 × 10^9^/L), a median presenting hemoglobin level (Hb) 13.3 g/dL (range: 9-22 g/dL), and a median presenting leukocyte count of 14.4 × 10^9^/L (range: 7-28 × 10^9^/L). Apart from five (11.1%) patients in whom the presenting symptomatology at presentation were not available for review, 25 (62.5%) were asymptomatic at diagnosis, four (10%) with bleeding, four (10%) with erythromelalgia, two (5%) with minor stroke, three (7.5%) with abdominal pain, and one each (2.5%) with blurred vision and weight loss. Of 39 patients with adequate follow-up information, five (12.8%) had myeloid transformations (MDS or AML) at the time of study. Of 40 patients with data on thrombosis, nine (22.5%) had thrombotic events. Apart from 5 patients with unknown JAK2 mutation status, 26 (65%) had JAK2 V617F mutation. (Table 1) The study has been approved by Institutional Review Board of Queen Mary Hospital with written informed consent.

**Table 1:** Patient demographic data and status of microRNA methylation

sex	age	Diagnosis	Symptoms at diagnosis	myeloid transformation	JAK2 V617F mutation	*miR-34b/c *	*miR-34a*	*miR-203*	*miR-124-1*
F	78	ET	Epigastric pain	No	yes	U	U	M	U
F	81	ET	Epigastric pain	No	yes	U	U	U	U
M	32	ET	erythromelalgia	No	yes	U	U	U	U
M	81	ET	N/A	No	yes	U	U	U	U
M	59	ET	N/A	N/A (No record)	yes	M	U	U	U
M	76	ET	Nil	No	yes	U	M	U	U
M	53	ET	Nil	MDS	yes	U	U	U	U
F	89	ET	Nil	N/A (No record)	yes	U	U	U	U
F	43	ET	Nil	N/A (No record)	yes	M	U	M	U
F	82	ET	Nil	No	yes	U	U	U	U
M	78	ET	Nil	No	yes	U	U	U	U
F	79	ET	Nil	No	yes	U	U	U	U
M	84	ET	Nil	AML	yes	U	U	U	U
F	74	ET	Nil	No	yes	M	U	M	U
F	56	ET	Nil	No	yes	U	U	U	U
M	60	ET	Nil	No	N/A	U	U	U	U
M	68	ET	Nil	AML	yes	U	U	U	U
M	39	ET	Nil	No	yes	U	U	U	U
F	79	ET	Weight loss	No	yes	U	U	U	U
F	60	ET	erythromelalgia	No	no	U	U	U	U
M	63	ET	Minor stroke (LUL numbness)	No	no	U	U	U	U
M	47	ET	erythromelalgia	No	no	U	U	U	U
F	62	ET	erythromelalgia, Headache	No	no	U	U	U	U
F	83	ET	N/A	N/A (No record)	no	U	U	M	U
F	32	ET	Nil	N/A (No record)	no	U	U	U	U
F	42	ET	Nil	No	no	M	U	U	U
M	85	ET	Nil	No	no	U	U	U	U
M	71	ET	Nil	No	no	U	U	U	U
F	28	ET	Nil	No	no	U	U	U	U
F	87	ET	Nil	No	no	U	U	U	U
M	48	ET	Nil	No	no	U	U	U	U
M	48	ET	Nil	No	no	U	U	U	U
M	41	ET	epistaxis	No	N/A	U	U	U	U
M	82	ET	N/A	No	N/A	U	U	U	U
F	41	PMF	Easy bruising	N/A (No record)	yes	U	U	U	U
M	67	PMF	Nil	No	yes	U	U	U	U
F	82	PMF	Nil	No	yes	U	U	U	U
F	42	PV	Minor stroke (RUL paraesthesia)	No	yes	U	U	U	U
M	48	PV	Gum bleeding	No	yes	U	U	U	U
F	75	PV	Nil	AML	yes	U	U	U	U
M	78	PV	Nil	No	yes	U	U	U	U
M	57	PV	Visual blurring (BRAO)	No	yes	U	U	U	U
M	44	PV	abdominal pain (splenic infarction)	No	no	U	U	U	U
M	71	PV	Gum bleeding	MDS	N/A	U	U	U	U
F	77	PV	N/A	No	N/A	U	U	U	U

Abbreviations: ET: essential thrombocythemia; PV: polycythemia vera; PMF: primary myelofibrosis; CVA: cerebrovascular acccident; BRAO: branch retinal vein occlusion; LUL: left upper limb; RUL: right upper limb; AML: acute myeloid leukemia; MDS: myelodysplastic syndrome; N/A: not available; U: unmethylated; M: methylated

### Cell lines and culture

MEG-01 and K-562 cells were kindly provided by Dr Mo Yang, Department of Paediatrics, Queen Mary Hospital, The University of Hong Kong, Hong Kong. HEL cells were obtained from Dr Dong-Er Zhang, Department of Pathology and Molecular Biology, Moores Cancer Center, University of California San Diego, USA. SET-2 cells were purchased from Deutsche Sammlung von Mikroorganismen und Zellkulturen GmbH (DMSZ) (Braunschweig, Germany). SET-2 was derived from ET at megakaryoblastic leukemic transformation. HEL was derived from AML M6. Both SET-2 and HEL cells carry JAK2 V617F mutation. MEG-01 and K-562 were derived from blastic transformation of patients with CML. Cell cultures were maintained in RPMI media 1640 (Invitrogen, Carlsbad, CA), supplemented with 10% (20% for SET-2) fetal bovine serum (Invitrogen, Carlsbad, CA), 50 U/ml penicillin, and 50 μg/ml streptomycin (Invitrogen, Carlsbad, CA) in a humidified atmosphere of 5% CO_2 _at 37°C.

### Methylation-specific polymerase chain reaction (MSP)

DNA was extracted from bone marrow samples at diagnosis and from cell lines by standard method. MSP for aberrant gene promoter methylation was performed as previously described [13,14]. Treatment of DNA with bisulfite for conversion of unmethylated cytosine to uracil (but unaffecting methylated cytosine) was performed with a commercially available kit (EpiTect Bisulfite Kit, Qiagen, Germany). Primers used for the methylated MSP (M-MSP) and unmethylated MSP (U-MSP) were shown in Table 2. DNA from normal bone marrow donors was used as negative control, while enzymatically methylated control DNA (CpGenome Universal Methylated DNA, Chemicon) was used as positive control in all the experiments. MSP was performed in a thermal cycler (9700, Applied Biosystems, Foster City, CA) with the following cycling conditions: 95°C for 5 minutes, specific cycles of 95°C for 30 seconds, specific annealing temperature for 30 seconds (Table 2), 72°C for 30 seconds, and a final extension of 10 minutes at 72°C. The MSP mixture contained 50 ng of bisulfite-treated DNA, 0.2 mM dNTPs, MgCl_2 _(Table 2), 10 pmol of each primer, 1 × PCR buffer, and 2.5 units of AmpliTaq Gold DNA Polymerase (Applied Biosciences, Foster City, CA) in a final volume of 25 μl. Ten microliters of PCR products were loaded onto 6% non-denaturing polyacrylamide gels, electrophoresed, and visualized under ultraviolet light after staining with ethidium bromide.

**Table 2:** MSP primer sequences and reaction conditions

Gene	Forward primer (5' - 3')	Reverse primer (5' - 3')	MgCl_2_/Tm/Cycles	Reference
*miR-34a*				
M-MSP	GGGGATGAGGATTAGGATTTC	ACAAAACGCATAAAAACGACG	1.5 mM/58°C/35	[9]
U-MSP	GGGGATGAGGATTAGGATTTT	CAAACAAAACACATAAAAACAACA	1.5 mM/58°C/35	
*miR-34b/c*				
M-MSP	ATTCGTTTCGTTTCGCGTTCGTTTC	CGACTACAACTCCCGAACGATCCG	2.0 mM/58°C/35	[34,35]
U-MSP	TTTTTATTTGTTTTGTTTTGTGTTTGTTTTG	CAACTACAACTCCCAAACAATCC	1.25 mM/56°C/38	
*miR-124-1*				
M-MSP	AAAGAGTTTTTGGAAGACGTC	AATAAAAAACGACGCGTATA	1.5 mM/55°C/35	[36]
U-MSP	AATAAAGAGTTTTTGGAAGATGTT	AAAAAAATAAAAAACAACACATATAC	2.0 mM/55°C/35	
				
*miR-203*				
M-MSP	GAGTATTTTCGGTTTAGACGAGAC	CCTTTTATACGACGCAACCG	1.5 mM/58°C/35	[37]
U-MSP	TTTGAGTATTTTTGGTTTAGATGAGAT	AACACCTTTTATACAACACAACCA	1.5 mM/58°C/35	

Abbreviations: Tm, annealing temperature; M-MSP, MSP for the methylated allele; U-MSP, MSP for the unmethylated allele

### 5-aza-2'-deoxycytidine (5-AzadC) treatment

HEL cells were homozygously methylated for *miR-34b/c*. Cells were seeded in six-well plates at a density of 1 × 10^6 ^cells/ml, and cultured with 1.5 μM of 5-AzadC for 7 days. Cells on day 0 and day 7 of treatment were harvested.

### RNA isolation and stem-loop reverse transcription-polymerase chain reaction (RT-PCR)

Total RNA was isolated using *mir*Vana™ miRNA Isolation Kit (Ambion, Austin, TX), according to the manufacturer's instructions. RT was performed using Taqman^® ^MicroRNA RT Kit and Taqman^® ^MicroRNA Assay Kit (Applied Biosciences, Foster City, CA), according to the manufacturer's instructions. Total RNA was reverse transcribed in 1 mM dNTPs, 50 U MultiScribe™ Reverse Transcriptase, 1X RT Buffer, 3.8 U RNase Inhibitor, and 1X stem-loop RT primer at the following thermal cycling condition: 16°C for 30 minutes, 42°C for 30 minutes, and 85°C for 5 minutes. Quantitative real-time PCR was performed using 1.33 μl of 1:15 diluted RT product in 1X Taqman^® ^Universal PCR Master Mix, and 1X Taqman^® ^Assay at 95°C for 10 minutes, followed by 40 cycles of 95°C for 15 seconds and 60°C for 1 minute. RNU48 was used as reference for data analysis using the 2^-ΔΔCt ^method [15]. Conventional RT-PCR for primary *miR-34a *was performed as previously described [9].

### Statistical analysis

Correlation between combined miR methylation status (i.e. those with methylation of any of these four miRs) with categorical variables (gender, MPN subtype, occurrence of myeloid transformation, development of thrombosis [either at diagnosis or after diagnosis of MPN] and presence of JAK2 V617F mutation) and continuous variables (age, presenting Hb, leukocyte and platelet counts) was computed by the Chi-square test (or Fisher Exact test) and Student's T-test. All p-values were two-sided.

## Results

### MSP

#### Controls

**Figure 1:**
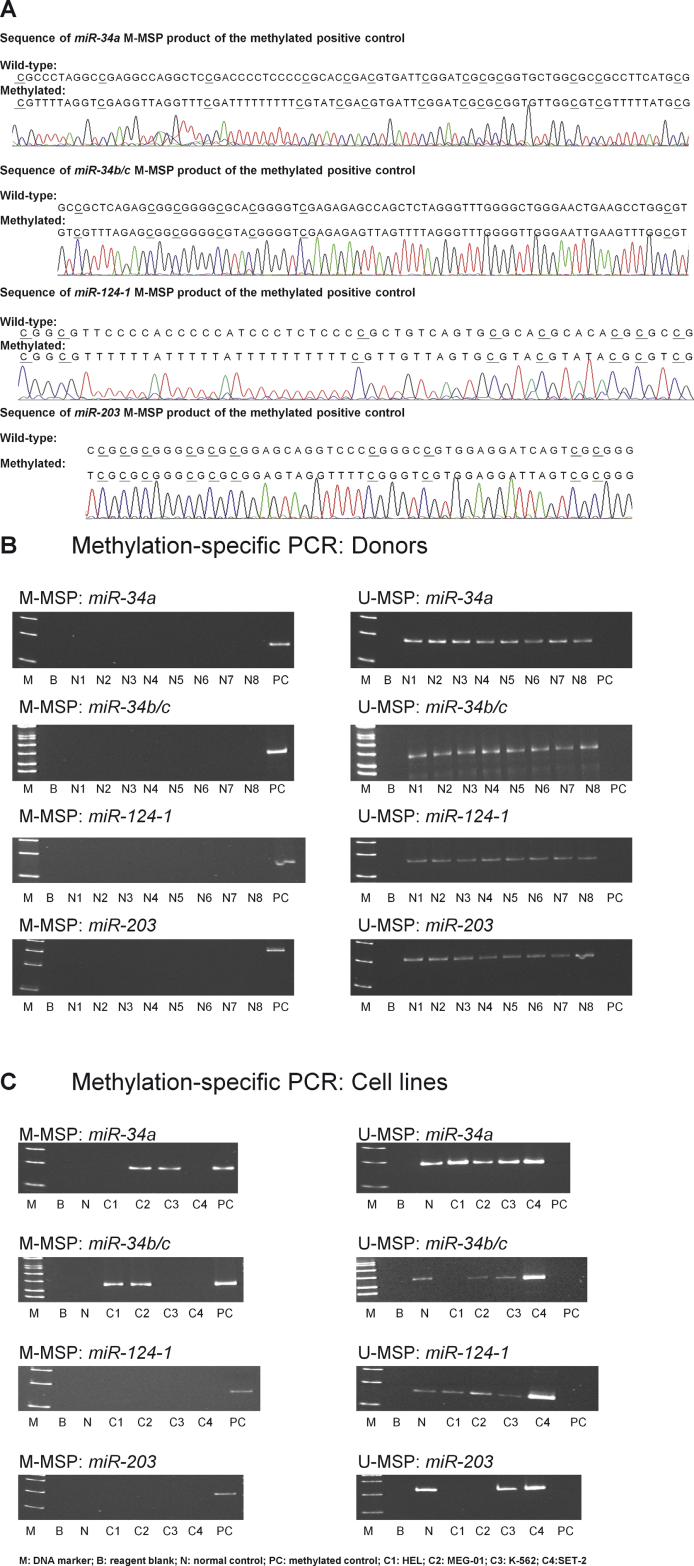
**Methylation of *miR-34a*, *miR-34b/c*, *miR-124-1 *and *miR-203***. A) Sequence analysis of the M-MSP product from bisulfite-treated positive control DNA showed that the cytosine [C] residues of CpG dinucleotides were methylated and remained unchanged, whereas all the other C residues were unmethylated and were converted to thymidine [T], confirming complete bisulfite conversion and MSP specificity. B) M-/U-MSP analysis showed that all the eight normal controls [N1-N8] were unmethylated. C) In the cell lines, MEG-01 and K-562 were hemizygously methylated for *miR-34a*; HEL was completely methylated, MEG-01 was hemizygously methylated for *miR-34b/c*; all the four cell lines were unmethylated for *miR-124-1*; K-562 and SET-2 were completely unmethylated for *miR-203*.

Direct sequencing of the M-MSP products from the methylated positive control confirmed the MSP specificity and complete bisulfite conversion, which methylated cytosine remained as cytosine upon sequencing (underlined) while unmethylated cytosine appeared as thymidine (Figure 1A). The positive and negative controls showed expected MSP results with normal DNA showing positive U-MSP but negative M-MSP amplification; and conversely, methylated control DNA showing negative U-MSP but positive M-MSP amplification. None of the 8 normal control marrows showed aberrant methylation of *miR-34a, -34b/c, -124-1 *or -*203 *(Figure 1B).

#### Cell lines

MSP analysis of the four cell lines showed that *miR-34a *was hemizygously methylated in MEG-01 and K-562 and completely unmethylated in HEL and SET-2. *miR-34b/c *was completely methylated in HEL, hemizygously methylated in MEG-01 and completely unmethylated in K-562 and SET-2. *miR-124-1 *was completely unmethylated in all the four cell lines. *miR-203 *was completely unmethylated in K-562 and SET-2. However, there was neither U- or M-MSP signals of *miR-203 *in both HEL and MEG-01, suggesting a possibility of homozygous deletion (Table 3; Figure 1C).

**Table 3:** MSP of miRs in cell lines

	*miR-34a*	*miR-34b/c*	*miR-124-1*	*miR-203*
Chromosomal location of miR	1p36	11q23	8p23	14q32
HEL	UU	MM	UU	-/-
MEG-01	UM	UM	UU	-/-
K-562	UM	UU	UU	UU
SET-2	UU	UU	UU	UU

UU: completely unmethylated; UM: hemizygously methylated; MM: homozygously methylated;

-/-: absence of both U- and M-MSP signals

#### Primary samples

**Figure 2:**
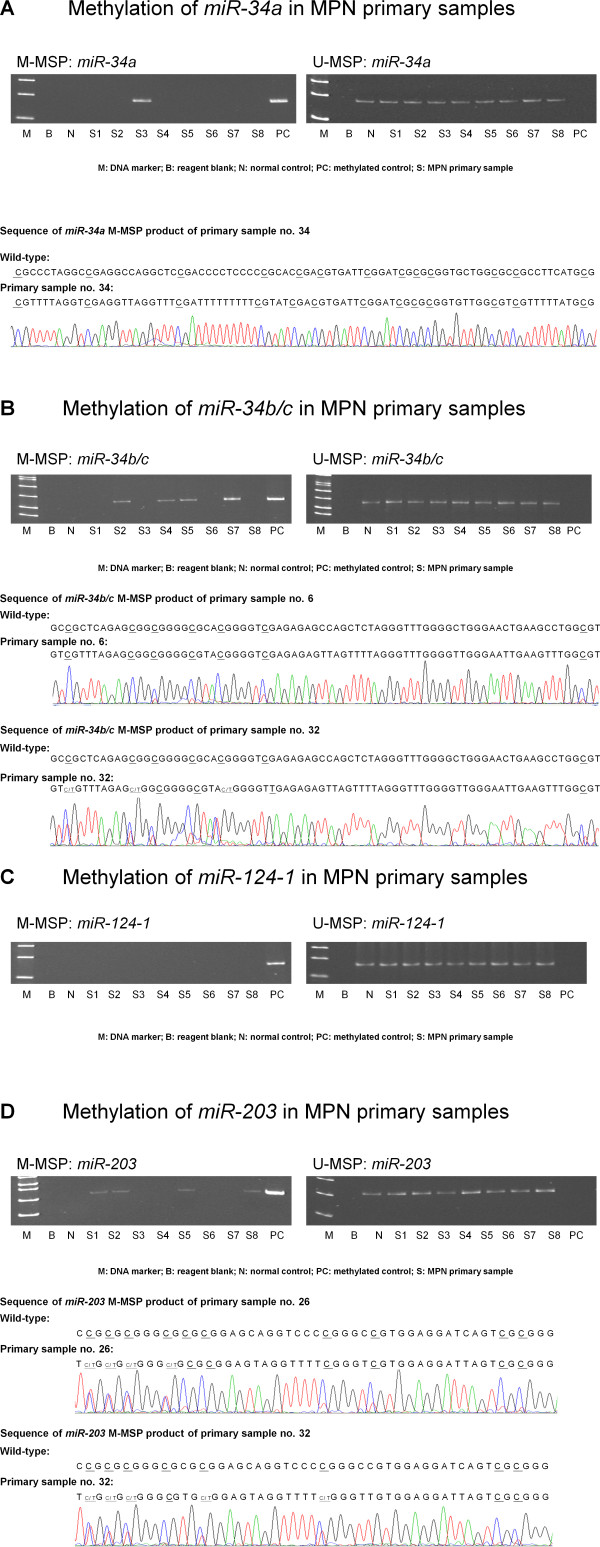
**Methylation of *miR-34a*, *miR-34b/c*, *miR-124-1 *and *miR-203 *in primary samples**. For each of these miRs, M-/U-MSP and sequencing of the M-MSP product from representative bisulfite-treated primary samples were shown. In the sequence analysis of the M-MSP product, cytosine [C] residues of CpG dinucleotides were methylated and remained unchanged, partially methylated C residues were denoted as [C/T], unmethylated C residues were converted into [T], whereas all the non-CpG C residues were unmethylated and were converted to thymidine [T], confirming complete bisulfite conversion and MSP specificity.

In the 45 primary bone marrow samples, *miR-34a *was methylated in one (2.2%), *miR-34b/c *in four (8.9%), *miR-203 *in four (8.9%) of patients but none had methylation of *miR-124-1 *(Figure 2; Table 1). Moreover, two (4.4%) had concomitant methylation of *miR-34b/c *and *-203 *but none had concomitant methylation of *miR-34a *and *-34b/c*. Direct sequencing of the methylated MSP products confirmed methylation of miRs in the primary samples (Figure 2). With regards to the MPN subtype, *miR-34a *methylation occurred in a patient with ET (2.9%), and *miR-203 *in four patients (11.8%) with ET. On the other hand, *miR-34b/c *methylation occurred in three patients (8.8%) with ET and one patient (12.5%) with PV. Overall, seven patients (15.6%) had methylation of at least one of the three miRs. *miR *methylation was not associated with age (p = 0.651), gender (p = 0.225), MPN subtype (p = 0.484), presenting Hb (p = 0.874), presenting leukocyte count (p = 0.969), presenting platelet count (p = 0.328), myeloid transformation (p = 0.99), thrombotic events (p = 0.311) or JAK2 V617F mutation (p = 0.99).

#### 5-AzadC treatment of HEL cells

**Figure 3:**
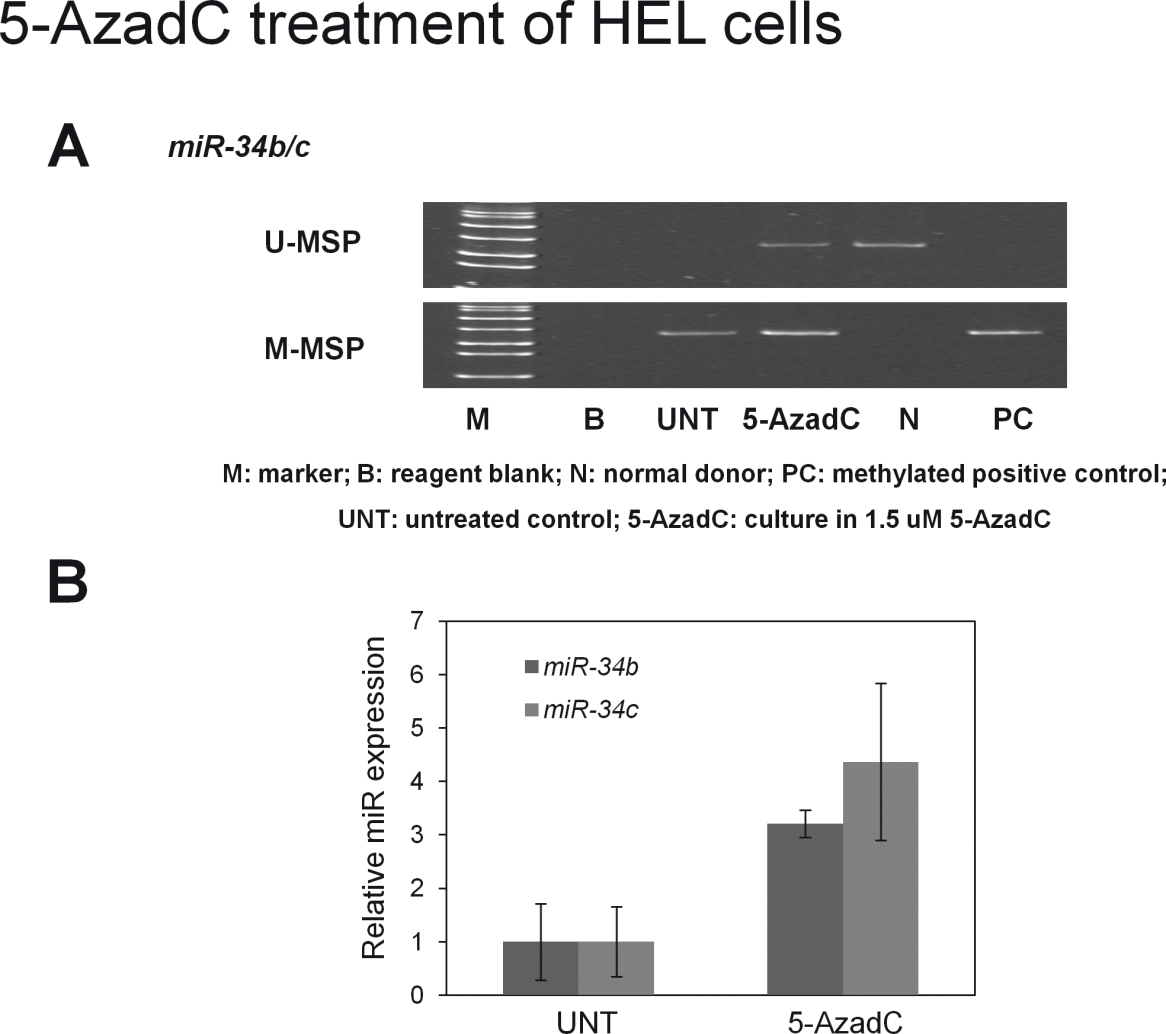
**Effect of 5-aza-2'-deoxycytidine (5-AzadC) treatment on HEL cells**. A) M-/U-MSP analysis of *miR-34b/c *promoter methylation status showed that 5-AzadC treatment led to progressive demethylation of *miR-34b/c *promoter in HEL cells. B) Stem-loop qRT-PCR analysis of mature *miR-34b *and *miR-34c *expression in HEL cells seven days after treatments. Error bar represents standard deviation.

Untreated HEL cells were homozygously methylated for *miR-34b/c *(Chr. 11q23). After 5-AzadC hypomethylation treatment, *miR-34b/c *U-MSP signal emerged, together with about 3-fold increase in expression of both mature *miR-34b *and *miR-34c *on day 7 as analyzed by stem-loop RT-PCR (Figure 3). On the other hand, *miR-34a *was unmethylated in HEL. By conventional RT-PCR of the primary transcript of *miR-34a*, *pri-miR-34a *was not constitutively expressed, and treatment with 5-AzadC did not lead to expression of primary *miR-34a *(Additional file 1).

## Discussion

Little information is available about the role of miRs in MPNs. Based on a literature search on the PubMed using keywords of "microRNA, methylation and myeloproliferative", no publication could be found. Therefore, this is likely the first report on methylation of miRs in MPN. In this study, we examined the methylation of *miR-34a*, *-34b/c*, *-124-1 *and *-203 *for a range of MPNs. In an attempt to identify miRs specifically involved in erythropoiesis, miR expression from *in vitro *expansion of erythroid cells derived from peripheral blood mononuclear cells were compared with controls, which showed *miR-451 *upregulation was specific to erythropoiesis [16]. On the other hand, during *in vitro *differentiation of megakaryocytes derived from CD34+ hematopoietic progenitors, downregulation of *miR-10a*, *-10b*, *-17*, *-20*, *-106 *and *-126 *was observed [17]. Moreover, when comparing the miR expression profiling of megakaryoblastic cell line with *in vitro *differentiated megakaryocytes, upregulation of *miR-99a*, *-101*, *-126*, and *-135 *was also found [17]. Therefore, the upregulation and downregulation of these specific miRs were associated with megakaryocytic and erythropoietic differentiation.

Despite that multiple TSGs were shown to be silenced by hypermethylation in AML [4,14,18-22], methylation of tumor suppressor genes was less frequently reported in MPN. Only recently, there were a few reports of methylation of the negative regulators of the JAK-STAT signaling pathway, *SOCS1, SOCS2 *and *SOCS3*, in Ph-ve MPN regardless of the JAK2 V617F mutation status [23-26].

Furthermore, based on previous work by us and others, *CDK6 *has been shown to be the target of multiple miRs including *miR-34a*, *-34b/c *and *-124-1 *[27,28]. Moreover, *CREB *is the target of *miR-34b *and *-203 *[10,29]. Finally, *ABL *is an additional target of *miR-203 *[12]. Consequently, epigenetic silencing of tumor suppressor *miR-34a*, *miR-34b/c*, *miR-124-1 *and *miR-203 *will confer proliferative advantage to the tumor cells [27-29]. In contrast to a previous report which showed *miR-203 *was methylated in Ph+ve but not Ph-ve MPN or leukemia, using MSP primers in the similar region [12], we demonstrated that *miR-203 *was hypermethylated in primary MPN samples, which was further verified by direct sequencing of the methylated MSP products. Therefore, it would appear that *miR-203 *methylation is involved in a wider spectrum of MPNs or leukemias, regardless of their Ph chromosome status. Finally, while two patients had concomitant methylation of *miR-203 *and *-34b/c*, none had concomitant methylation of *miR-34a *and *-34b/c*, both transcriptional targets of p53, and hence avoiding duplication of tumor suppressor gene inactivation of the same pathway.

In HEL and MEG-01 cells, both U- and M-MSP signals of *miR-203 *were absent, which might be due to the following possibilities: (1) sample DNA degradation, (2) inappropriate PCR condition, or (3) homozygous deletion of the region. Since simultaneous U-MSP analysis of the same DNA sample for *miR-34a*, *miR-34b/c*, and *miR-124-1 *promoter successfully generated the U-MSP signals, hence the absence of MSP signals for *miR-203 *in HEL and MEG-01 cells could not be explained by a poor DNA quality. Moreover, as *miR-203 *U-MSP was successful in all the other samples including cell lines, normal controls, patient samples and methylated positive control, inappropriate MSP conditions appears unlikely. Therefore, the absence of both M- and U-MSP signals in HEL and MEG-01 cells might be caused by deletions of the region. However, karyotypic data of HEL and MEG-01 cells did not reveal homozygous deletion of 14q32, and hence whether absence of MSP amplification of *miR-203 *might be due to microdeletion of this region requires further study [30]. Furthermore, hypomethylation treatment of the HEL cells, which was homozygously methylated for the *miR-34b/c*, a microRNA cluster localized to 11q23, showed significant re-expression of mature *miR-34b *and *miR-34c*. This finding is consistent with that both *miR-34b *and *miR-34c *are under the promoter regulation of the same CpG island. By contrast, *miR-34a*, another member of the *miR-34 *family localized to 1p36, was not constitutively expressed. Moreover, hypomethylating treatment did not lead to expression of primary *miR-34a*, suggesting additional mechanism, possibly histone modification, in the regulation of *miR-34a *expression [9]. Furthermore, in addition to showing miR silencing in cell line, it is important to show the correlation of miR methylation and miR expression in the primary sample. In this connection, we shall collect both DNA and RNA from diagnostic bone marrow samples in the future.

In order to identify miRs that are methylated specifically at the time of transformation to AML or MDS, and hence implicated for pathogenesis of myeloid transformation, ideally one should analyze the paired marrow samples at both diagnosis and leukemic/myelodysplastic transformation. This is exemplified by our recent publication in the study of epigenetic inactivation of *miR-34b/c *methylation in myeloma, in which we showed that while *miR-34b/c *is not methylated at diagnosis, it is frequently methylated at the time of relapse or disease progression. This is as evidenced by the significantly more frequent methylation of *miR-34b/c *of myeloma samples at relapse in patients with both diagnostic and relapse marrow samples[31].

Finally, unlike the association of TSG with clinical parameters, such as the association of *CDKN2B *and *WIF1 *methylation with high presenting leukocyte count in acute promyelocytic leukemia [32,33], methylation of these miRs did not correlate with demographic, presenting blood counts, JAK2 V617F mutation or complications including thrombosis and myeloid transformations.

## Conclusion

This is the first report of miR hypermethylation in MPNs. *miR-203 *hypermethylation is not specific to Ph+ve leukemias but also present in Ph-ve MPNs. *miR-34b/c *methylation was associated with reversible miR silencing. There was no correlation of miR methylation with clinical demographic data or outcome.

## Competing interests

The authors declare that they have no competing interests.

## Authors' contributions

All authors read and approved the final manuscript. CSC is responsible for design of study, interpretation of data and writing manuscript. TSW is responsible for provision of study material. KYW and TKF are responsible for conduction of experiment. HGD is responsible for provision of cell lines. KFW is responsible for provision of patients' material.

## Supplementary Material

Additional file 1 **Effect of 5-aza-2'-deoxycytidine (5-AzadC) treatment on HEL cells**. 5-AzadC treatment of HEL cells. Click here for file
