# Supplementary figures and images for: Methylation of miR-34a, miR-34b/c, miR-124-1 and miR-203 in Ph-negative myeloproliferative neoplasms
Source: J Transl Med. 2011 Nov 14;9:197. doi: 10.1186/1479-5876-9-197 (PMC3283527; doi:10.1186/1479-5876-9-197)

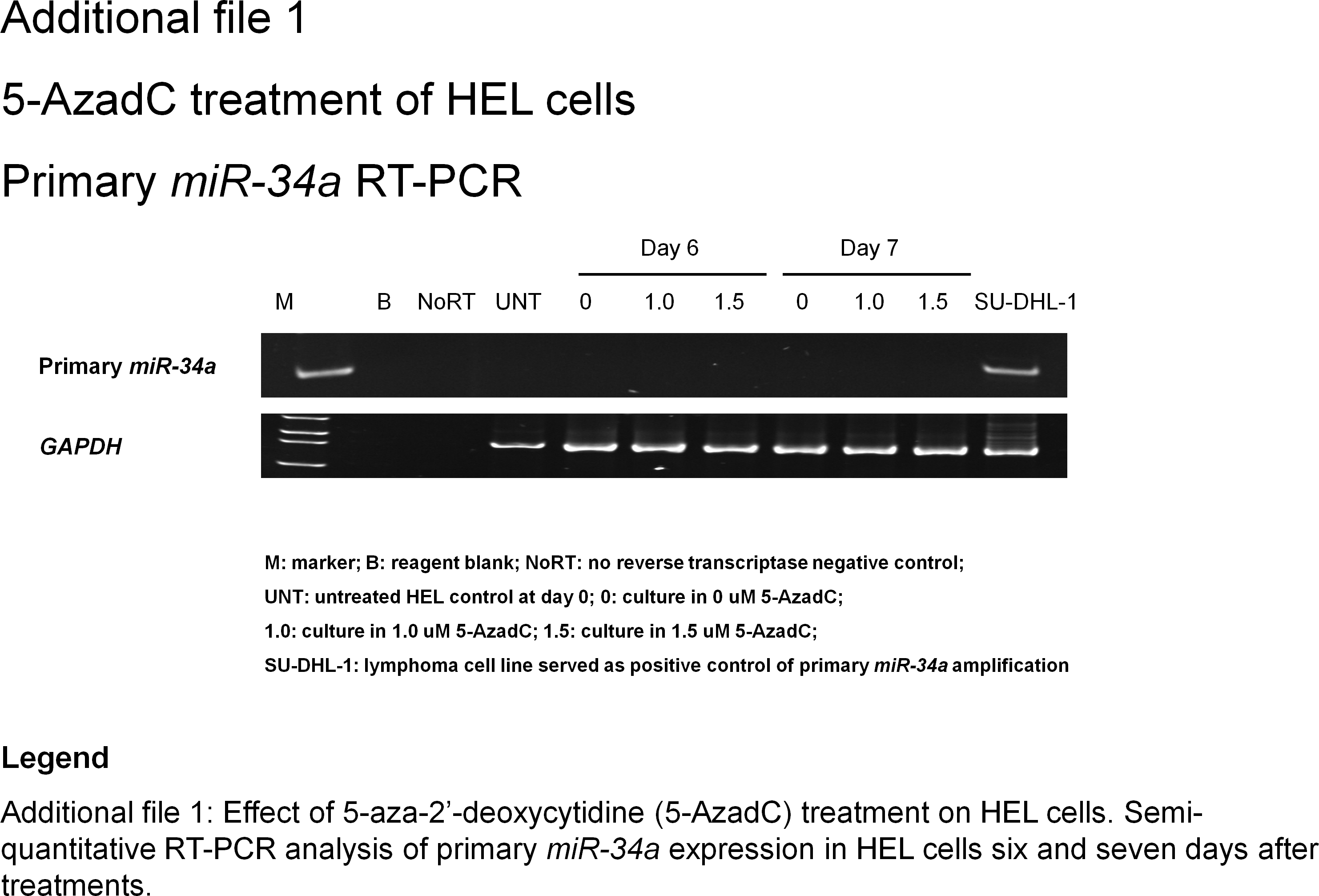

Supplement: Additional file 1 — Effect of 5-aza-2'-deoxycytidine (5-AzadC) treatment on HEL cells. 5-AzadC treatment of HEL cells. [file 1479-5876-9-197-S1.TIFF]
